# Supplementary material for: Integrated network pharmacology and molecular docking approaches to reveal the synergistic mechanism of multiple components in Venenum Bufonis for ameliorating heart failure
Source: PeerJ. 2020 Oct 30;8:e10107. doi: 10.7717/peerj.10107 (PMC7605218; doi:10.7717/peerj.10107)
Supplement: Supplemental Information 1 [file peerj-08-10107-s001.docx]

**Table S1**

*In vivo* constituents of VB.

| NO. | Chemical name | Chemical structure | Reference |
| --- | --- | --- | --- |
| P1 | Resibufogenin  (C_24_H_32_O_4_) |  | Xia et al. 2010 |
| P2 | Bufalin  (C_24_H_34_O_4_) |  | Xia et al. 2010 |
| P3 | 19-oxo-bufalin  (C_24_H_32_O_5_) |  | Xia et al. 2010 |
| P4 | Desacetylcinobufagin  (C_24_H_32_O_5_) |  | Ning et al. 2010 |
| P5 | Marinobufagin  (C_24_H_32_O_5_) |  | Xia et al. 2010 |
| P6 | Resibufaginol  (C_24_H_32_O_5_) |  | Xia et al. 2010 |
| P7 | 19-hydroxybufalin  (C_24_H_34_O_5_) |  | Xia et al. 2010 |
| P8 | Desacetylbufotalin  (C_24_H_34_O_5_) |  | Xia et al. 2010 |
| P9 | Gamabufotalin  (C_24_H_34_O_5_) |  | Xia et al. 2010 |
| P10 | Telocinobufagin  (C_24_H_34_O_5_) |  | Xia et al. 2010 |
| P11 | 19-oxo-desacetylcinobufagin  (C_24_H_30_O_6_) |  | Xia et al. 2010 |
| P12 | Arenobufagin  (C_24_H_32_O_6_) |  | Xia et al. 2010 |
| P13 | Hellebrigenin  (C_24_H_32_O_6_) |  | Xia et al. 2010 |
| P14 | Cinobufagin  (C_26_H_34_O_6_) |  | Tao et al. 2017 |
| P15 | Bufotalin  (C_26_H_36_O_6_) |  | Tao et al. 2017 |
| P16 | Cinobufaginol  (C_26_H_34_O_7_) |  | Xia et al. 2010 |
| P17 | Cinobufotalin  (C_26_H_34_O_7_) |  | Liang et al. 2008 |
| M1 | 3-oxo-resibufogenin  (C_24_H_30_O_4_) |  | Zhu et al. 2013 |
| M2 | 3-epi-bufalin  (C_24_H_32_O_4_) |  | Xia et al. 2010 |
| M3 | 3-oxo-desacetylcinobufagin  (C_24_H_30_O_5_) |  | Ning et al. 2010; He et al. 2012 |
| M4 | 5β-hydroxyresibufogenin  (C_24_H_32_O_5_) |  | Zhu et al. 2013 |
| M5 | 3,14,15-trihydroxy-bufa-20,22-dienolide  (C_24_H_34_O_5_) |  | Zhu et al. 2013 |
| M6 | 6α-hydroxybufalin  (C_24_H_34_O_5_) |  | Xin et al. 2016 |
| M7 | 5β-hydroxybufalin  (C_24_H_34_O_5_) |  | Xin et al. 2016 |
| M8 | 1,5-dihydroxyresibufogenin  (C_24_H_32_O_6_) |  | Zhu et al. 2013 |
| M9 | 1-hydroxyldesacetylcinobufagin  (C_24_H_32_O_6_) |  | Ning et al. 2010 |
| M10 | 5,16-dihydroxy-resibufogenin  (C_24_H_32_O_6_) |  | Zhu et al. 2013 |
| M11 | 5-hydroxyldesacetylcinobufagin  (C_24_H_32_O_6_) |  | He et al. 2012 |
| M12 | 12,16-dihydroxy-resibufogenin  (C_24_H_32_O_6_) |  | Zhu et al. 2013 |
| M13 | 12-hydroxyldesacetylcinobufagin  (C_24_H_32_O_6_) |  | He et al. 2012 |
| M14 | 1α,5β-dihydroxybufalin  (C_24_H_34_O_6_) |  | Xin et al. 2016 |
| M15 | 3,5,14,15-tetrahydroxyl-bufa-20,22-dienolide  (C_24_H_34_O_6_) |  | Zhu et al. 2013 |
| M16 | 5β, 6α-dihydroxybufalin  (C_24_H_34_O_6_) |  | Xin et al. 2016 |
| M17 | 1,5-dihydroxyldesacetylcinobufagin  (C_24_H_32_O_7_) |  | Ning et al. 2010 |
| M18 | 1,12β-dihydroxydesacetylcinobufagin  (C_24_H_32_O_7_) |  | He et al. 2012 |
| M19 | 2,5-dihydroxyldesacetylcinobufagin  (C_24_H_32_O_7_) |  | Ning et al. 2010 |
| M20 | 3-oxo-cinobufagin  (C_26_H_32_O_6_) |  | He et al. 2012 |
| M21 | 5-hydroxyl-cinobufagin  (C_26_H_34_O_7_) |  | He et al. 2012 |
| M22 | 12-hydroxyl-cinobufagin  (C_26_H_34_O_7_) |  | He et al. 2012 |
| M23 | 3-sulfate-bufalin  (C_24_H_34_O_7_S) |  | Miyashiro et al. 2008 |
| M24 | 1,12β-dihydroxycinobufagin  (C_26_H_34_O_8_) |  | He et al. 2012 |
| M25 | 3-sulfate-marinobufagin  (C_24_H_32_O_8_S) |  | Miyashiro et al. 2008 |
