# Supplementary figures and images for: Integrated network pharmacology and molecular docking approaches to reveal the synergistic mechanism of multiple components in Venenum Bufonis for ameliorating heart failure
Source: PeerJ. 2020 Oct 30;8:e10107. doi: 10.7717/peerj.10107 (PMC7605218; doi:10.7717/peerj.10107)

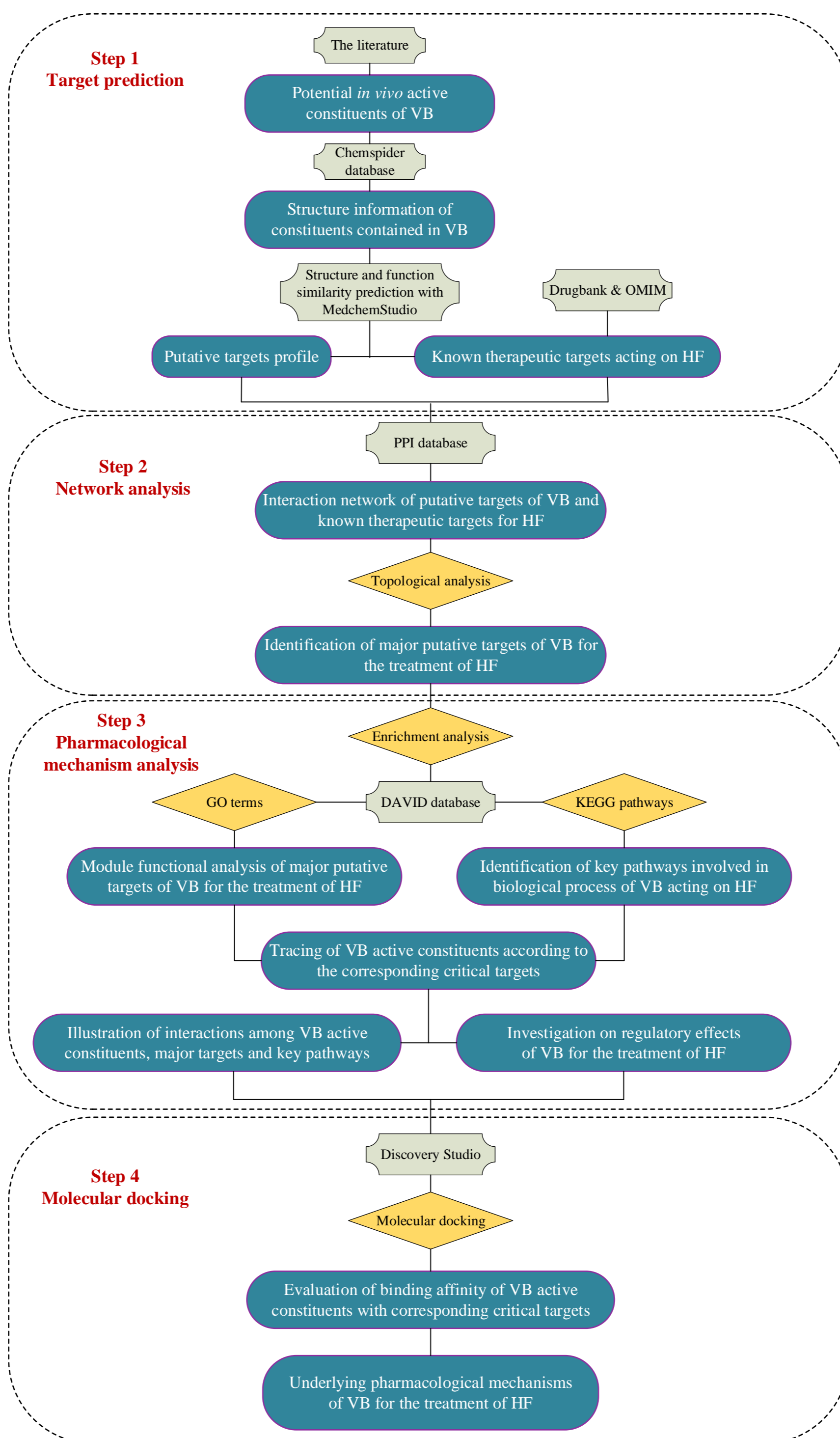

Supplement: Supplemental Information 7 [file peerj-08-10107-s007.pdf]
